# Opposite Auxin Dynamics Determine the Gametophytic and Embryogenic Fates of the Microspore

**DOI:** 10.3390/ijms241311177

**Published:** 2023-07-06

**Authors:** Yolanda Pérez-Pérez, María Teresa Solís, Alfonso Albacete, Pilar S. Testillano

**Affiliations:** 1 Pollen Biotechnology of Crop Plants Group, Biological Research Center Margarita Salas, CIB-CSIC, Ramiro de Maeztu 9, 28040 Madrid, Spain; yperez@cib.csic.es; 2 Department of Genetics, Microbiology and Physiology, Complutense University of Madrid, 28040 Madrid, Spain; msolis03@ucm.es; 3 Department of Plant Nutrition, Center for Edaphology and Applied Biology of Segura, CEBAS-CSIC, Campus Universitario de Espinardo, 30100 Murcia, Spain; alfonsoa.albacete@carm.es

**Keywords:** auxin, pollen development, microspore embryogenesis, cell reprogramming, kynurenine

## Abstract

The microspore can follow two different developmental pathways. In vivo microspores follow the gametophytic program to produce pollen grains. In vitro, isolated microspores can be reprogrammed by stress treatments and follow the embryogenic program, producing doubled-haploid embryos. In the present study, we analyzed the dynamics and role of endogenous auxin in microspore development during these two different scenarios, in *Brassica napus.* We analyzed auxin concentration, cellular accumulation, the expression of the *TAA1* auxin biosynthesis gene, and the *PIN1-like* efflux carrier gene, as well as the effects of inhibiting auxin biosynthesis by kynurenine on microspore embryogenesis. During the gametophytic pathway, auxin levels and *TAA1* and *PIN1-like* expression were high at early stages, in tetrads and tapetum, while they progressively decreased during gametogenesis in both pollen and tapetum cells. In contrast, in microspore embryogenesis, *TAA1* and *PIN1-like* genes were upregulated, and auxin concentration increased from the first embryogenic divisions. Kynurenine treatment decreased both embryogenesis induction and embryo production, indicating that auxin biosynthesis is required for microspore embryogenesis initiation and progression. The findings indicate that auxin exhibits two opposite profiles during these two microspore developmental pathways, which determine the different cell fates of the microspore.

## 1. Introduction

The plasticity of plant cells, specifically their competency to regenerate embryos through in vitro culture, has been extensively exploited for several decades with the purpose of plant propagation, breeding, and conservation of numerous species of interest. This has resulted in significant applications in the fields of agriculture, forestry, and genetic resource preservation [1]. In vitro embryogenesis has been successfully induced in a diverse variety of somatic cell types, including haploid microspores, which are capable of acquiring totipotency and embryogenic competence through the influence of specific inducting factors. This ultimately results in the formation of complete embryos [2]. In vivo, within the anther, microspores follow a strictly regulated gametophytic developmental pathway to produce mature pollen grains capable of fertilization. During this process, the different layers and tissues that compose the anther also play varying roles, with the tapetal cells being particularly important. The tapetum is the anther tissue in closest contact with the developing pollen grains. It is formed by a single layer of cells and acts as an important nurturing tissue that provides nutrients and regulatory molecules to the sporogenous tissue for microspore formation and pollen grain development. Among other functions, tapetum is involved in the synthesis of enzymes that release microspores in a tetrad by degrading the callose wall. It also plays an important role in exine coverage and pollenkit formation [3]. It is known that with the progression of pollen development, the tapetum undergoes a crucial process of programmed cell death (PCD) [4]. On the other hand, in vitro, the microspore, at the responsive stage of vacuolated microspore, can be reprogrammed towards an embryogenic pathway, producing haploid and doubled-haploid embryos and plants. This alteration in the in vivo developmental progression requires specific stress treatments depending on the plant species [5,6]. This process, known as stress-induced microspore embryogenesis, is the most widely used method for producing doubled-haploid plants. It allows for the rapid creation of new isogenic lines and represents a source of new genetic variability fixed in homozygous plants, obtained in a single generation [7,8,9,10,11,12,13]. Although stress-induced microspore embryogenesis is an important tool in breeding programs, its efficiency is still limited in many economically important species. This limitation arises from the fact that embryogenesis initiation and progression involve a complex network of factors whose roles are still largely unknown [2,14,15]. Several factors, including stress signaling, hormones, and epigenetic mechanisms, have been proposed as essential components in the acquisition of totipotency and the induction of embryogenesis in microspores [2], as well as in somatic embryogenesis from other cell types [16,17].

Hormonal regulation, specifically auxin, has been described as one of the determinant factors involved in the regulation of the embryogenesis process in many species [14,18]. Auxins are recognized as the most significant group of phytohormones involved in plant growth, playing a pivotal role in the regulation of cellular division and differentiation [19]. Many studies have described the involvement of the predominant form of auxin in plants, indole-3-acetic acid (IAA), in numerous developmental processes, such as apical dominance of shoots [20], stem and coleoptile growth [21], formation and differentiation of leaf vascular tissue [22], root meristem development [23], and lateral root formation [24]. There are also studies describing the involvement of IAA in the process of zygotic embryogenesis, from the early stages [25] to the embryo polarization and differentiation [18,26]. However, despite the increased knowledge in recent years about hormonal regulation in various developmental processes, the precise role of auxin in initiating and promoting in vitro embryogenesis is still not well understood. It is well described that auxin action primarily depends on its local biosynthesis and differential distribution within plant tissues, which is primarily regulated by its directional transport between cells [27]. The main pathways for auxin biosynthesis, transport, and signaling have been extensively studied over the past few decades in the model species *Arabidopsis thaliana* [28,29]. Various routes have been identified for the synthesis of auxin, with the indole-3-pyruvic acid (IPA) pathway being the principal one in most eudicot and monocot species [30,31]. The IPA pathway is a two-step pathway where the enzymes tryptophan aminotransferase of Arabidopsis 1 (TAA1) and tryptophan aminotransferase–related 1 and 2 (TAR1, TAR2) catalyze the conversion of tryptophan amino acid into IPA. In a second reaction, flavin monooxygenases of the YUCCA family (YUC) convert IPA into IAA [31,32]. A useful technique for studying the involvement of TAA1/TAR-dependent auxin biosynthesis is the use of a small molecule called kynurenine (Kyn), which competitively inhibits the activity of TAA1/TAR [33]. The use of Kyn has shown inhibitory effects on auxin biosynthesis and consequently causes defects in many plant developmental processes related to auxin [34,35]. Since auxin biosynthesis occurs locally in the plant, a transport mechanism is necessary to distribute it to its targets in different plant tissues. This process is primarily carried out through a polar cell-to-cell transport mechanism known as polar auxin transport (PAT), which is mainly mediated by the transporters called PIN-FORMED (PIN) [27,36,37,38]. Among the eight PIN proteins described in *Arabidopsis*, PIN1 plays a central role in the directional control of PAT during zygotic embryo development [21,39].

Usually, data regarding the role of auxin in somatic embryogenesis are obtained through in vitro approaches, often using systems that involve the addition of exogenous auxin concentrations to the culture media [40,41,42]. In contrast, in *Brassica napus*, microspore embryogenesis occurs from isolated microspores under culture conditions where exogenous auxin is not added at any point [43,44]. This aspect makes the in vitro embryogenesis system of *B. napus* highly suitable for studying the role of endogenous auxin during microspore reprogramming and embryo development. Specifically, the isolated microspore culture of *B. napus* is considered the primary model system for stress-induced microspore embryogenesis in dicotyledonous plants [8]. In this system, cell reprogramming to initiate the new developmental process is typically induced by a stress condition involving a mild heat treatment of 32 °C [43].

In the present study, we investigated the dynamics of endogenous auxin during in vivo gametophytic development and compared it with its profile during in vitro microspore reprogramming and embryogenesis initiation and progression in *B. napus*. We analyzed changes in endogenous IAA accumulation using the technique of HPLC coupled to tandem mass spectrometry. We also employed immunofluorescence assays with anti-IAA antibodies and performed confocal microscopy analyses to examine the spatial distribution of auxin. Additionally, we analyzed the expression patterns of the auxin biosynthesis and transport genes (*BnTAA1* and *BnPIN1*). Furthermore, functional analyses were conducted by adding the auxin biosynthesis inhibitor kynurenine to the in vitro culture to assess its effect on embryogenesis efficiency.

## 2. Results

### 2.1. Endogenous Auxin Accumulation and Distribution Pattern during Pollen Development and Microspore Embryogenesis

We performed immunofluorescence assays using anti-IAA antibodies to analyze the presence of auxin at the cellular level during the two developmental pathways of the microspore: pollen development and microspore embryogenesis. First, we examined the main stages of these processes at a microscopic level.

**Figure 1:**
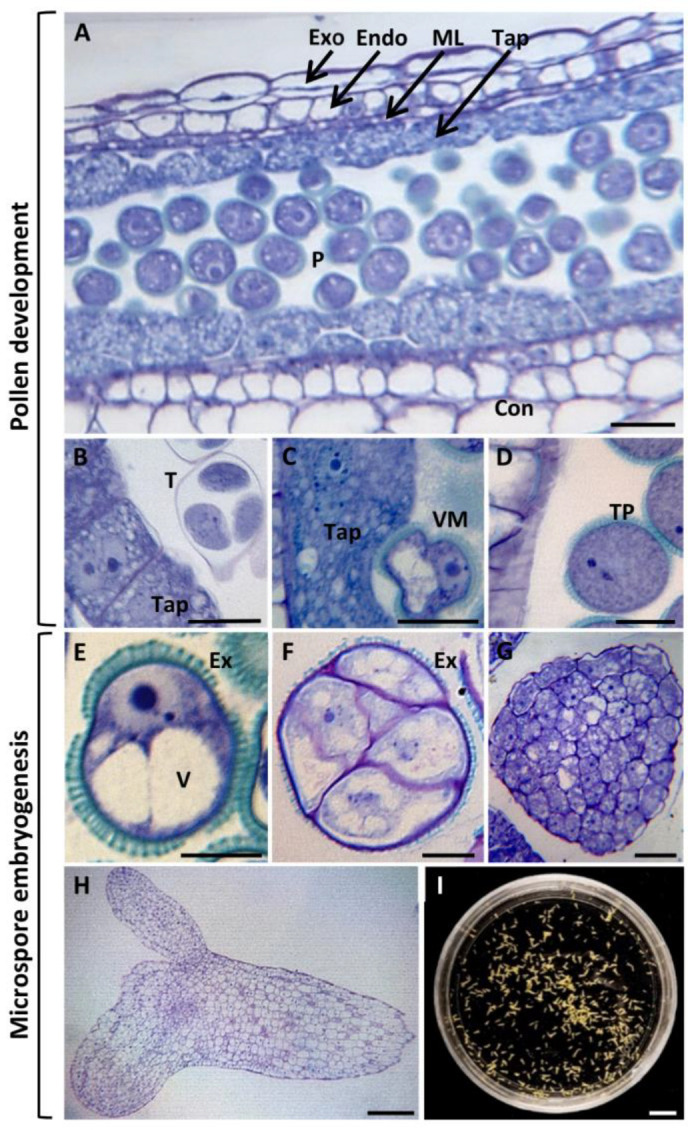
Main stages of in vivo pollen development (**A**–**D**) and stress induced-microspore embryogenesis (**E**–**I**) of *Brassica napus*. (**A**–**H**) Micrographs of semithin sections stained by toluidine blue observed in bright field. (**A**) Panoramic view of a longitudinal anther section, showing the different cell layers that form the anther structure. (**B**–**D**) Anther at different developmental stages: (**B**) anther at the tetrad stage with very active tapetum tissue; (**C**) anther at the stage of vacuolated microspore with tapetum in early programmed cell death (PCD); (**D**) anther at the stage of mature pollen, where tapetum has been completely degraded; (**E**) isolated vacuolated microspore, at the beginning of the microspore embryogenesis culture; (**F**) proembryo, still surrounded by exine, first structural sign of embryogenesis initiation; (**G**) globular embryo; (**H**) cotyledonary embryo; (**I**) panoramic view of a Petri dish of microspore embryogenesis culture after 30 days, showing cotyledonary embryos. Exo: exothecium; Endo: endothecium; ML: medium layer; P: pollen; Tap; tapetum; T: tetrad; VM: vacuolated microspore; TP: tricellular (mature) pollen; Ex: exine; V: vacuole. Bars represent (**E**,**F**) 10 µm, (**B**–**D**,**G**) 20 µm, (**A**,**H**) 50 µm, and (**I**) 1 cm.

Pollen development takes place inside the anthers, which consist of several cell layers. From the outermost to the innermost zone, we observed the exothecium, endothecium, medium layer, and tapetum (Figure 1A). Within the pollen sac, after the male meiosis, a tetrad of microspores is produced, surrounded by a temporary wall of callose (Figure 1B). At this stage, the tapetum appears as a thick layer of large polygonal cells with dense cytoplasm. Subsequently, the microspores are released from the tetrad and undergo development, forming vacuolated microspores characterized by a large vacuole (Figure 1C). During this stage, the tapetum starts undergoing programmed cell death (PCD), with the cells losing their polygonal shape, experiencing cytoplasm shrinkage and vacuolization, and exhibiting nuclei compaction and chromatin condensation. Following an asymmetric division, the vacuolated microspore gives rise to a bicellular pollen grain, with a small generative cell located within the cytoplasm of a larger vegetative cell. After mitosis of the generative cell, the tricellular pollen is formed, consisting of a vegetative cell and two sperm cells (Figure 1D). At this final stage, the tapetum has completely degraded and is no longer observed (Figure 1D).

The natural developmental pathway of the microspore can be changed in vitro under specific conditions. In the in vitro system, an isolated vacuolated microspore (Figure 1E) can be reprogrammed through the application of a heat stress treatment (32 °C), resulting in the acquisition of totipotency and the initiation of the embryogenic pathway. The reprogrammed microspores underwent several divisions that produced proembryos (Figure 1F), which are still surrounded by the exine microspore wall. Then, cell divisions continued and increased cell proliferation, giving rise to globular embryos (Figure 1G). These globular embryos further develop into heart, torpedo, and differentiated cotyledonary embryos (Figure 1H), which are formed after 30 days in culture (Figure 1I).

To analyze the subcellular localization of endogenous IAA during both developmental programs, we analyzed changes in the distribution patterns of endogenous auxin using immunofluorescence assays with a specific IAA monoclonal antibody. Image acquisition was performed using confocal laser scanning microscopy, with consistent excitation and emission capture settings applied to all immunofluorescence preparations. This ensured that fluorescence signal intensity could be accurately compared among different developmental stages throughout each process.

**Figure 2:**
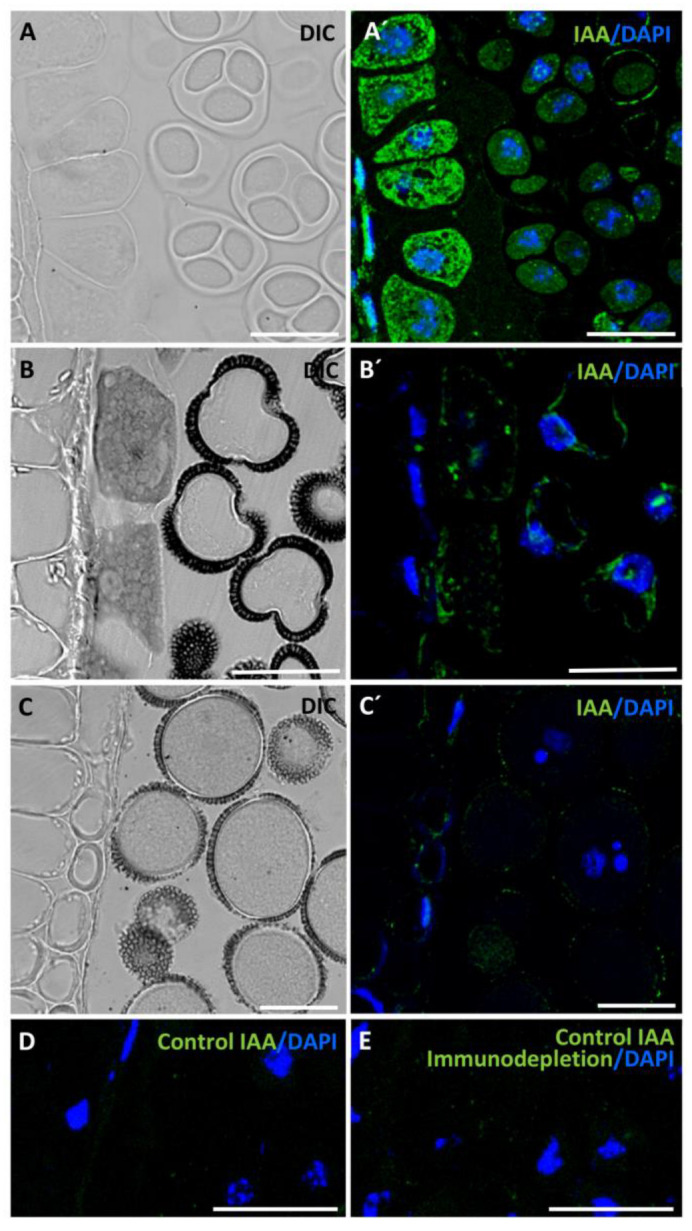
Immunolocalization of IAA during in vivo pollen development. Confocal microscopy images showing IAA immunofluorescence signal (green), DAPI staining of nuclei (blue), and Nomarski’s differential interference contrast (DIC). (**A**,**A′**) Anther at the tetrad stage with very active tapetum tissue, (**B**,**B′**) anther at the stage of vacuolated microspore with tapetum in early programmed cell death (PCD), (**C**,**C′**) anther at the stage of mature pollen, without tapetum. (**D**,**E**) Negative control experiments of IAA immunofluorescence at the tetrad stage by eliminating the primary antibody (**D**) and using the anti-IAA antibody preblocked with IAA (immunodepletion) (**E**). Bars represent 20 µm.

During in vivo pollen development, at the tetrad stage, both microspores and tapetum exhibited an intense immunofluorescence signal localized in the cytoplasm and the nuclei of the cells (Figure 2A′). In later stages, vacuolated microspores showed an immunofluorescence signal of mid intensity in the thin peripheral layer of the cytoplasm, with no signal observed in the large central vacuole of the cell. Additionally, the fluorescence signal in tapetum cells also decreased (Figure 2B′). At the late developmental stage of mature pollen, the results showed a very weak or undetectable signal within the pollen grains, and the tapetum layer had practically disappeared at this stage (Figure 2C′).

To support the specificity of the antibody and the immunolocalization results, negative controls by eliminating the IAA antibody and by immunodepletion assays were performed. In both cases, the negative controls did not show significant labelling in the samples (Figure 2D,E).

**Figure 3:**
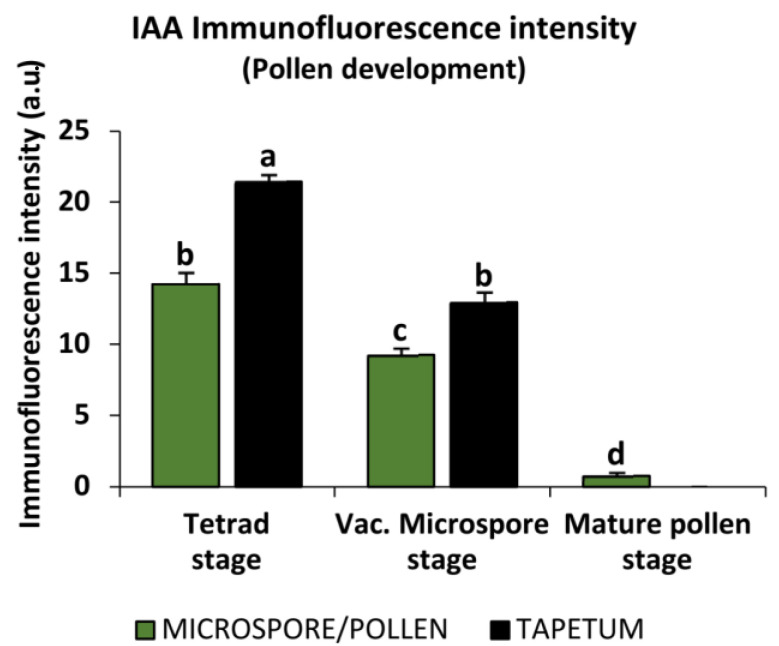
Quantification of IAA immunofluorescence signal intensity at the cellular level of both pollen and tapetum tissue during pollen development. Histograms represent the level of immunofluorescence intensity, in arbitrary units, as measured by ImageJ software tools over confocal maximum projection images at different microspore and tapetum developmental stages (tetrads/active tapetum, vacuolated microspores/tapetum in early PCD, and mature pollen grains/tapetum degraded). Columns represent the mean fluorescence intensity (±SEM) of images from at least three immunofluorescence experiments and three biological replicates. Different letters indicate significant differences according to ANOVA and Tukey’s test at *p* ≤ 0.05.

The intensity of the IAA signal was quantified using the ImageJ software (version V1.8.0), with separate analysis conducted for microspores or pollen grains and their corresponding tapetum cells. The results demonstrated a significant reduction in IAA signal during the progression of in vivo pollen development. A high signal intensity was observed at the tetrad stage in both tetrad and tapetum cells, followed by a lower signal in vacuolated microspores and tapetum cells at this stage. The signal intensity was much lower in mature pollen grains, where tapetum cells could not be measured due to their advanced PCD state at this late stage of pollen development (Figure 3).

**Figure 4:**
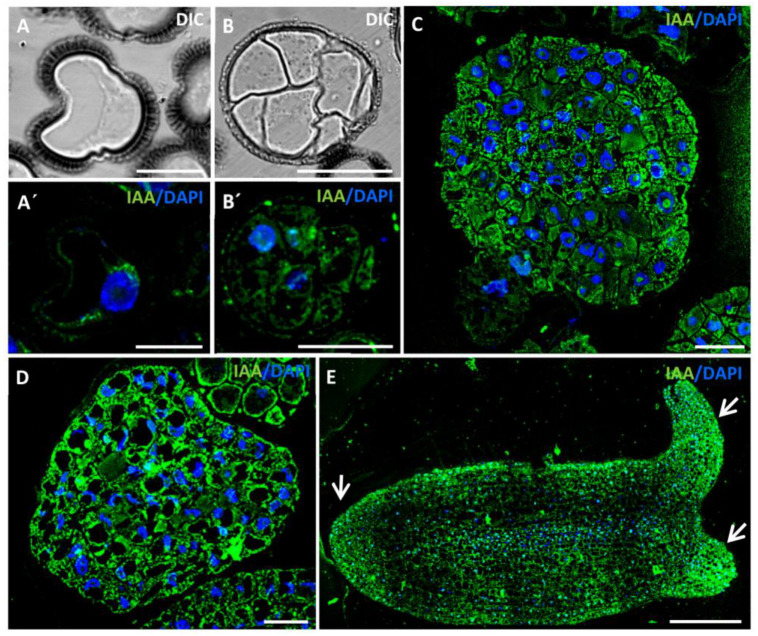
Immunolocalization of IAA during stress-induced microspore embryogenesis. Confocal microscopy images showing IAA immunofluorescence signal (green), DAPI staining of nuclei (blue), and Nomarski’s differential interference contrast (DIC). (**A**,**A′**) Isolated vacuolated microspore at the beginning of the culture. (**B**,**B′**) Proembryo. (**C**) Globular embryo conserving the suspensor structure. (**D**) Heart-shaped embryo. (**E**) Cotyledonary embryo. Arrows point to the apical zone in cotyledons and root apical meristem, regions with higher IAA accumulation. Bars represent (**A**,**A′**) 10 µm, (**B**,**B′**,**C**,**D**) 25 µm, and (**E**) 75 µm.

Analyses of the microspore embryogenesis pathway revealed that, before induction, the isolated vacuolated microspore exhibited a very low IAA signal (Figure 4A′). However, after induction, an increase in IAA signal was observed in the proembryo cells (Figure 4B′). Subsequent developmental stages, including globular (Figure 4C), heart (Figure 4D), and cotyledonary embryos (Figure 4E), exhibited a gradual increment in auxin signal intensity with a heterogeneous distribution. During the globular embryo stage, some embryos developed a suspensor, a characteristic structure observed during in vivo embryogenic development that connects the zygotic embryo to surrounding tissues during early seed development. Suspensor cells displayed a very low auxin signal compared with the other cells that constitute the embryo (Figure 4C). The highest intensity of the IAA signal was observed in cotyledonary embryos, with a specific distribution pattern showing the highest signal in the root meristem and the apical zone of cotyledons (Figure 4D). The negative controls performed during the microspore embryogenesis stages did not show any labelling.

**Figure 5:**
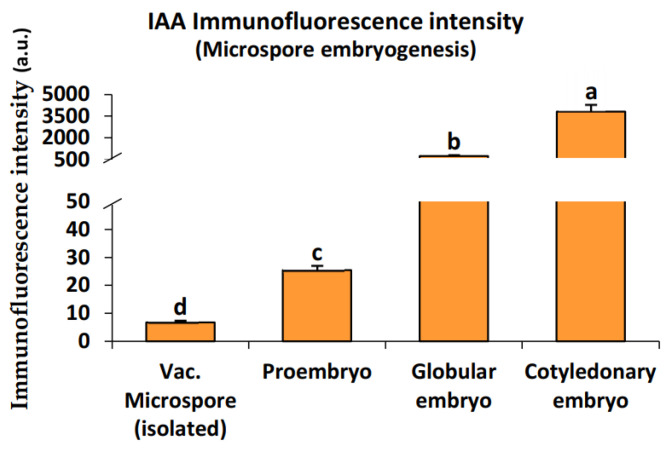
Quantification of IAA immunofluorescence signal intensity at the cellular level during stress-induced microspore embryogenesis. Histograms represent the level of immunofluorescence intensity, in arbitrary units (a.u.), as measured by ImageJ software tools over confocal maximum projection images at different developmental stages (isolated vacuolated microspores, proembryos, globular embryos, and cotyledonary embryos). Columns represent the mean fluorescence intensity (±SEM) of images from at least three immunofluorescence experiments and three biological replicates. Different letters indicate significant differences according to ANOVA and Tukey’s test at *p* ≤ 0.05.

The quantitative image analysis revealed a highly significant increase in the immunofluorescence intensity of endogenous auxin during the progression of in vitro microspore embryogenesis progression. The vacuolated microspores exhibited a low auxin signal, which increased notably after the induction of embryogenesis and throughout the subsequent stages of embryo development (Figure 5).

### 2.2. Endogenous Auxin Concentration during Pollen Development and Microspore Embryogenesis

**Figure 6:**
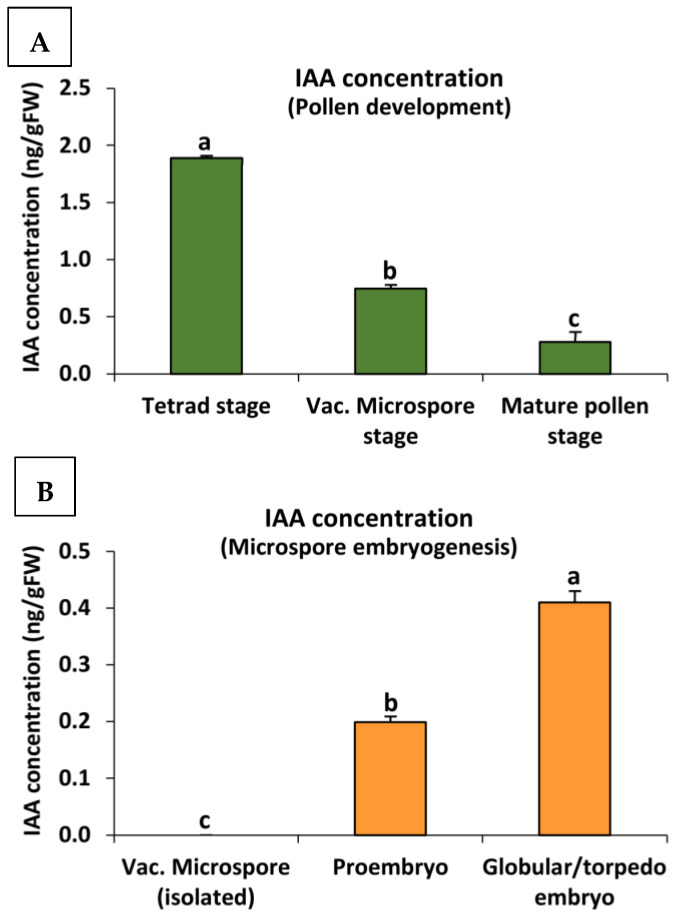
Quantification of endogenous IAA concentration during in vivo pollen development (**A**) and stress-induced microspore embryogenesis (**B**). Histograms represent the level of endogenous IAA, in nanograms per gram of fresh weight, as measured by using the technique of liquid chromatography electrospray ionization tandem mass spectrometry (LC/ESI–MS/MS), using anthers and culture samples at different developmental stages for both microspore developmental programs. Each column represents the mean (±SEM) of at least three biological and three technical replicates. Different letters indicate significant differences according to ANOVA and Tukey’s test at *p* ≤ 0.05.

The analysis of endogenous IAA concentration using the technique of liquid chromatography electrospray ionization tandem mass spectrometry (LC/ESI–MS/MS) provided insights into the changes in auxin levels during different developmental stages of both the gametophytic and embryogenic pathways. In the case of pollen development, the results showed a high concentration of endogenous IAA at the early stage of the tetrad, which gradually decreased as the microspores developed, reaching the lowest levels in mature pollen (Figure 6A).

On the other hand, in vitro microspore embryogenesis displayed a different pattern of endogenous IAA concentration. Initially, the isolated vacuolated microspores exhibited low levels of auxin before reprogramming. However, upon embryogenesis initiation, there was an increase in endogenous IAA levels, which further increased as the embryogenesis process progressed. The advanced stages of globular and torpedo embryos showed the highest levels of auxin concentration (Figure 6B).

### 2.3. Expression Patterns of Auxin Biosynthesis and Transport Genes BnTAA1 and BnPIN1-like during Pollen Development and Microspore Embryogenesis

**Figure 7:**
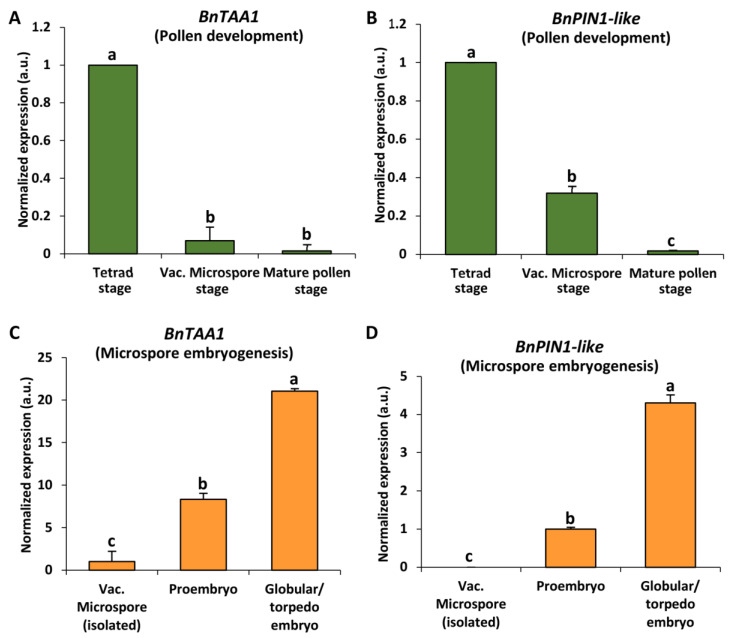
Expression patterns of the genes *BnTAA1* (auxin biosynthesis gene) (**A**,**C**) and *BnPIN1-like* (auxin transport gene) (**B**,**D**) during in vivo pollen development (**A**,**B**) and stress-induced microspore embryogenesis (**C**,**D**). Histograms show relative changes of mRNA levels at different developmental stages for both microspore developmental programs, normalized to the tetrad stage in case of pollen development and to isolated vacuolated microspore in case of microspore embryogenesis, as determined by RT-qPCR. Each column represents the mean of at least three biological and three technical replicates. Bars indicate the standard error of the mean (SEM). Different letters indicate significant differences among stages within the expression of each gene according to ANOVA and Tukey’s tests at *p* < 0.05.

The expression analysis of auxin-related genes, specifically auxin biosynthesis gene *BnTAA1* and auxin transport gene *BnPIN1-like*, was performed by qRT-PCR during the two microspore developmental pathways. The results revealed similar expression patterns for both auxin biosynthesis (*BnTAA1*) and transport (*BnPIN1-like*) genes within each developmental program: both genes were downregulated during the pollen developmental program (Figure 7A,B) and upregulated during microspore embryogenesis progression (Figure 7C,D).

During pollen development, *BnTAA1* showed high expression in anthers at the tetrad stage and almost nondetectable levels at later developmental stages, in vacuolated microspore and mature pollen (Figure 7A). Additionally, *BnPIN1-like* showed higher expression in the tetrad, which reduced by less than half at the stage of vacuolated microspore, and with almost no expression in the most advanced stage of mature pollen (Figure 7B).

In contrast, during microspore embryogenesis progression, the studied genes showed very low expression (in case of *BnPIN1-like*) (Figure 7D) or no expression (in case of *BnTAA1*) (Figure 7C) in isolated vacuolated microspore, before embryogenesis induction. However, a significant increase was observed in the proembryo stage, accompanying embryogenesis initiation (Figure 7C,D). At more advanced stages, in globular/torpedo embryos, results revealed a much higher expression of the two auxin-related genes, approximately fourfold than proembryos.

These results indicate the activation of auxin biosynthesis during microspore embryogenesis initiation and its progressive upregulation during embryo development, concomitant with the activation of polar auxin transport (PAT). In contrast, the gametophytic pathway exhibited a decrease in auxin levels as it progressed.

### 2.4. Effect of the Inhibition of Auxin Biosynthesis on Microspore Embryogenesis Initiation and Somatic Embryo Formation

**Figure 8:**
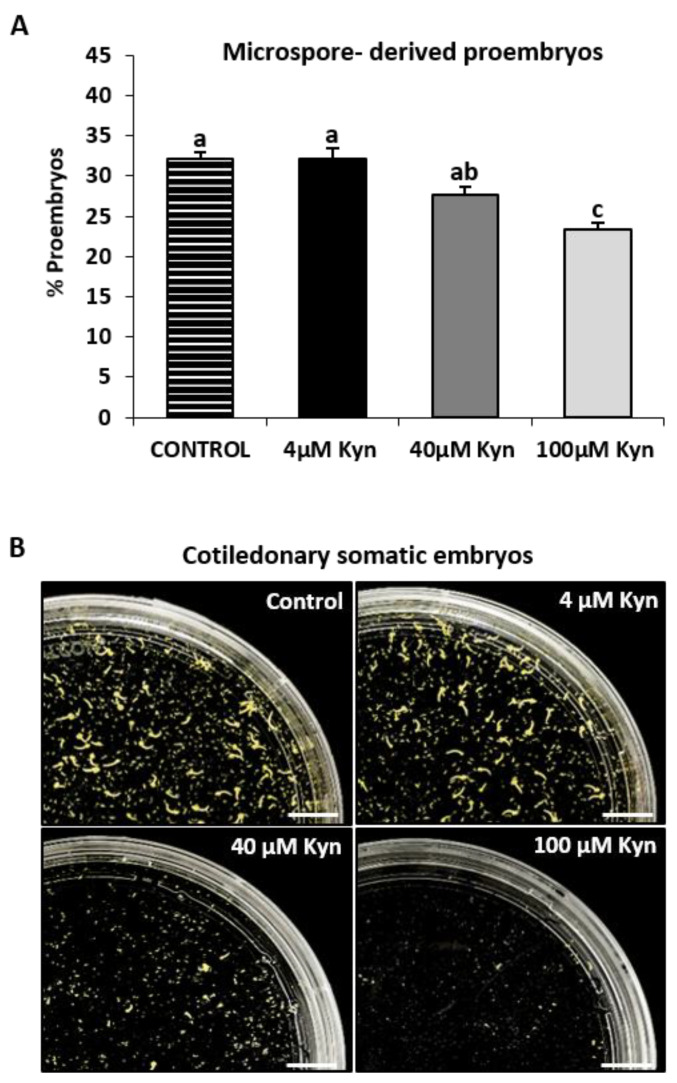
Effect of kynurenine (inhibitor of auxin biosynthesis) treatments on stress-induced microspore embryogenesis. (**A**) Quantification of microspore embryogenesis initiation, measured as the percentage of proembryos obtained in untreated cultures and cultures treated with kynurenine at different concentrations (4, 40, and 100 µM). Histograms show the mean percentage of proembryos in untreated (control) and treated cultures. Each column represents the mean of three biological and three technical replicates. Bars in columns indicate the standard error of the mean (SEM). Different letters on columns indicate significant differences according to ANOVA and Tukey’s tests at *p* < 0.05. (**B**) Plates showing the microspore-derived embryos produced after 30 days in untreated and treated cultures with 4, 40, and 100 µM kynurenine. Bars represent: 1 cm.

To analyze the potential involvement of endogenous auxin in microspore embryogenesis, the inhibitor of auxin biosynthesis, kynurenine, was added in the culture media at concentrations of 4, 40, and 100 µM immediately after placing the microspores in culture. The effect of the inhibitor on embryogenesis induction and progression was examined. First, the effect of kynurenine on embryogenesis induction was assessed by quantifying the percentage of proembryos (the first morphological sign of embryogenesis initiation) in both untreated (control) and treated cultures. The cultures treated with kynurenine exhibited a significant reduction in the percentage of proembryos, with approximately a 25% decrease observed at a concentration of 100 µM compared with the control cultures (Figure 8A). No significant differences were observed at lower concentrations tested.

To evaluate the effect of the inhibitor on embryogenesis progression, the production of differentiated cotyledonary embryos was assessed in control and treated cultures after 30 days of culture. At this time point, the control cultures exhibited a significant number of cotyledonary embryos (Figure 8B). However, in cultures treated with the inhibitor, a dose-dependent decrease in the number of differentiated embryos was observed, particularly at concentrations of 40 and 100 µM (Figure 8B). In fact, very few differentiated structures were observed in cultures treated with 100 µM concentration of kynurenine (Figure 8B).

These results indicate that the inhibition of auxin biosynthesis affected both embryogenic induction and subsequent embryo development, suggesting an important role of auxin in both the initiation and progression of microspore embryogenesis in *Brassica napus*.

## 3. Discussion

The aim of this study was to compare the dynamics of endogenous auxin during the two developmental pathways of the microspore, in vivo pollen development and stress-induced microspore embryogenesis, and its possible role during initiation and the whole process of microspore embryogenesis in *Brassica napus*, a model system for induced cellular reprogramming in plants. There are several studies that describe the relevant role that auxin plays during floral development [45,46,47]. However, to date, research that reports on the involvement of this hormone in pollen development is very limited. Likewise, the evidence indicates that hormonal regulation plays a crucial role in in vitro somatic embryogenesis from various explants, with auxin being particularly important for reprogramming somatic cells towards embryogenesis [16,48,49]. Most somatic embryogenesis systems typically rely on the application of exogenous hormones, particularly the synthetic auxin 2,4-dichlorophenoxyacetic acid (2,4-D), often in combination with stress treatments, to initiate induction [16,17,50,51]. In contrast, many microspore embryogenesis systems, such as in *B. napus*, do not require exogenous auxin as a trigger for embryogenesis, because just a temporary stress treatment can induce the transition towards embryogenic fate [5,52]. Although the involvement of auxin in early somatic embryogenesis has been documented [2,53], the role of this hormone throughout the entire microspore embryogenesis process is not yet fully understood.

### 3.1. The Gametophytic Fate of the Microspore: Auxin Decreases during Pollen Development

In vivo, the development of the male gametophyte is closely linked to the dynamics and differentiation of the anther tissues, especially the tapetum. Previous studies have demonstrated that pollen grain formation is accompanied by metabolic and morphological changes in tapetal cells [54]. In a recent study conducted in *Arabidopsis* [55], the use of the synthetic auxin response promoter DR5: GUS revealed the presence of auxin throughout pollen development, with higher levels detected in microspores and lower levels in tricellular and mature pollen where anthesis has already taken place. Furthermore, using the same technique, auxin signaling activity was detected in tapetal cells during early stages of floral development, specifically during the microspore stage prior to pollen grain formation [55]. In our study, the localization of auxin was performed using immunofluorescence assays with the monoclonal antibody anti-IAA, which demonstrated specificity in our analyses as well as in previous studies with various negative and positive controls [56,57]. This technique enabled precise localization of the IAA signal in different anther tissues during pollen development, providing detailed information on auxin distribution and levels based on fluorescent signal intensity. The observed auxin accumulation patterns during *B. napus* pollen development are consistent with the findings reported in the aforementioned *Arabidopsis* study, indicating high auxin levels during early stages of pollen development in both tapetal cells and microspores at the tetrad stage, followed by a gradual decrease in auxin levels as development progress. These immunolocalization results also correlate with the endogenous IAA concentration results obtained in anthers in this study using the LC/ESI–MS/MS technique.

Previous studies have provided evidence of the importance of auxin in tapetal cells for proper pollen grain and stamen development [58,59]. Other studies in *Arabidopsis* have demonstrated the involvement of auxin in both early and late stages of stamen development [47]. During early stages, the expression of the auxin biosynthesis genes YUC2 and YUC6, which are associated with tapetum differentiation, activity, and fate, has been observed. Similarly, our immunolocalization results have shown a high presence of auxin in tapetal cells during early stages, supporting the role of auxin in the tapetum of *B. napus* during early stages of anther development. These findings are also consistent with the transcription levels of the auxin biosynthesis gene *BnTAA1*, which exhibits high expression levels during early stages of development, followed by a decrease in transcripts levels as microspore differentiation progresses. A study conducted by Sundberg and Østergaard (2019) [47] also demonstrated the presence of auxin in later stages of stamen development in *Arabidopsis*, suggesting its involvement in stamen filament elongation and anther dehiscence. However, our findings do not show the presence of auxin in the pollen grain or other anther tissues during later stages. This leads us to speculate that auxin may be localized in the filament at this stage, a tissue that was not analyzed in our study. Other studies have indicated that both auxin biosynthesis and transport are crucial during early and late stages of stamen development. A study in *Arabidopsis* [60] revealed the involvement of various members of the PIN-FORMED (PIN) transporter family in the initial stages of stamen formation and development in both monocotyledonous and dicotyledonous plants. These findings are consistent with our gene expression analyses of *BnPIN1-like*, which demonstrated high expression in the early stages of pollen development, followed by a decrease in later stages. This expression pattern aligns with the results obtained for the *BnTAA1* biosynthesis gene, as well as with the immunolocalization and endogenous concentration of IAA. Our results indicate that auxin, along with the activation of its biosynthesis and transport pathway, is necessary during early stages of pollen development in *B. napus*. However, as pollen maturation progresses, there is a negative regulation of both auxin biosynthesis and transport, as well as a decrease in cellular auxin concentration in both pollen and tapetal cells.

### 3.2. The Change of Microspore Fate: Stress-Induced Cell Reprogramming Involve the Activation of Auxin Biosynthesis and Transport

For many years, exogenous hormones have been utilized to induce in vitro somatic embryogenesis and subsequent embryo development. Among all the known growth regulators, auxin is the most employed in culture media for somatic embryogenesis. This hormone plays a crucial role in both direct embryogenesis processes, to initiate embryo development, and indirect embryogenesis, for the formation of proembryogenic masses and the subsequent development of somatic embryos. However, the mechanisms that regulate the triggering of these processes in the presence of auxin remain largely unknown [2,16,48,61]. In various species where indirect embryogenesis is induced, high levels of auxin have been observed during the proliferation of proembryogenic masses and early somatic embryo development [41,62,63,64,65]. Similarly, in direct embryogenesis, specifically microspore embryogenesis, higher auxin levels have been observed in early embryos compared with nonresponsive microspores that have not undergone the stress treatment [43,56,66]. In *Brassica napus*, microspore embryogenesis is induced and progresses without the addition of exogenous auxin to the culture media at any time. Our results have revealed a high concentration of auxin in proembryos during the early embryogenic divisions, suggesting a role for this hormone in the activation of either cell reprogramming and/or proliferation that follows immediately. Studies conducted on the somatic embryogenesis of other species have shown an increase in endogenous IAA levels throughout the process [17,57,61,67,68,69,70,71], indicating the activation of de novo auxin biosynthesis, polar transport, and signaling pathways, evidenced by high expression levels of the biosynthesis genes *YUC*, *TAA1*, and *TAR2*; the auxin polar transport gene *PIN1*; and the signaling genes *Aux*/*IAA* and *ARF*. In *Arabidopsis* zygotic embryogenesis, an increase in auxin levels was observed in ovule tissues after fertilization [72], as well as in zygotic embryo cells from the very beginning of their formation [18,73]. Several studies have demonstrated a correlation between IAA levels and localization during somatic embryogenesis and in vivo zygotic embryogenesis in several species [43,74,75]. Regarding the distribution pattern of auxin in the zygotic embryo, several in vitro studies have detected a peak signal in the apical meristem of the stem and root in maize and barley [56,76,77], as well as in the apical zone of cotyledons in several dicotyledonous plants [21,25,43], coinciding with regions of maximum auxin biosynthesis.

The results of the present study demonstrate that in *B. napus*, at advanced stages, the microspore-derived embryo exhibits an auxin localization pattern with a large accumulation in the same meristematic regions as the zygotic embryo. Specially, there is a significant accumulation of auxin in the most apical zone of the cotyledons where leaf primordia will develop, as well as in the root meristem zone that will give rise to the radicle after embryo germination. Once the embryogenic pathway is activated, the subsequent embryo development involves a further increase in endogenous auxin levels. The results obtained in our work show an upregulation of the biosynthesis gene *BnTAA1* and an increase in the immunofluorescence signal and concentration of IAA during embryo differentiation. In the case of *B. napus* microspore embryogenesis, which initiates without surrounding extraembryonic tissue and in a growth-regulator-free culture medium, our results suggest that the heat stress treatment used to reprogram microspores can induce an endogenous auxin response, increasing its biosynthesis and intracellular levels, which will determine the initiation of the new developmental pathway and cell fate of the microspores. Additionally, the increase in auxin levels in early proembryos may be associated with the activation of proliferative activity in both reprogrammed microspores and proembryo cells. When discussing the action and availability of auxin, it is important to consider its cell-to-cell transport, highlighting the significant role of the family of transporter proteins PIN-FORMED (PIN) [38]. Several studies have demonstrated the auxin transport in early zygotic embryogenesis stages from the suspensor cells to the rest of the embryo by the transporter PIN7, while PIN1 would distribute auxin evenly among the developing embryo cells [27,78]. Moreover, these transporter proteins, along with PIN4, are involved in stablishing auxin gradients necessary for subsequent cell differentiation in advanced stages of embryo development [27,39]. In the present work, gene expression analysis of *BnPIN1-like* during microspore embryogenesis in rapeseed showed increased transcription at the beginning of embryogenesis in proembryos, and even higher expression levels in differentiating embryos. These results align with the observed polarization of auxin in cotyledonary embryos using immunofluorescence techniques, where a differential auxin accumulation pattern is observed, particularly with higher levels in the apical region of cotyledons and the basal zone of the embryo, areas with higher proliferation activity in the embryo. In several species, it has been also observed that during the early stages of zygotic embryogenesis, there is an auxin flow from the suspensor to the rest of the embryo [18,73,75]. Our results indicate that some embryos derived from microspores in the globular or heart stage can develop while retaining the suspensor structure. Interestingly, these embryos exhibit very little fluorescence signal in the suspensor compared with the rest of the actively proliferating cells that form the embryo. All these results indicate that the synthesis and localization dynamics of auxin in microspore embryogenesis in rapeseed resemble those of zygotic embryogenesis.

### 3.3. Inhibition of Auxin Biosynthesis by Kynurenine Impaired Microspore Embryogenesis Initiation and Progression

For years, the importance of auxin transport and action in plant development has been extensively studied, performing functional analyses with known auxin inhibitors such as N-1-naphthylphthalamic acid (NPA), an inhibitor of auxin transport, and α-(*p*-chlorophenoxy)-isobutyric acid (PCIB), an inhibitor of auxin action. These inhibitors have been shown to cause defects in the normal vegetative and reproductive growth, including embryogenesis [30,40,57,79,80]. However, efficient and specific inhibitors of auxin biosynthesis have only become available recently [70]. In the present work, functional analyses were carried out by adding kynurenine in the in vitro cultures, a small molecule that selectively inhibits the enzymatic activity of TAA1/TAR, directly affecting the main auxin biosynthesis pathway [33]. The results demonstrated the negative effect of the treatment at two crucial points in the embryogenesis process. On the one hand, cultures treated with the inhibitor showed a lower percentage of proembryos after applying the inductive heat stress compared with control cultures. On the other hand, as the culture progressed, the kynurenine treatment also reduced microspore embryo differentiation. These results indicate that de novo auxin biosynthesis plays a crucial role at the beginning of the culture, in microspore reprogramming, and in the subsequent embryo development and differentiation during microspore embryogenesis in *B. napus*. In a previous study conducted by our group on the monocot plant *Hordeum vulgare* (where microspore embryogenesis is induced by cold stress treatment), a similar effect of kynurenine was observed. The inhibitor reduced embryogenesis induction at early stages and decreased the number of coleoptilar embryos produced at advanced stages [56]. Our results, together with those obtained in microspore embryogenesis of *H. vulgare* [56], indicate that activation of auxin biosynthesis is required for stress-induced microspore embryogenesis in various species, regardless of the type of applied stress (cold or heat), suggesting common hormonal regulation pathways during the process.

### 3.4. Opposite Auxin Dynamics Determine the Two Distinct Microspore Developmental Pathways and Cell Fate

**Figure 9:**
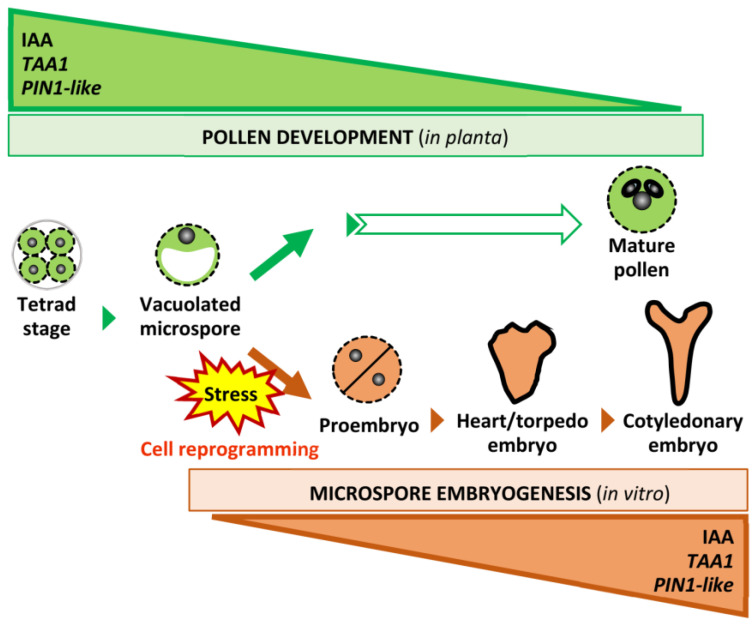
Scheme representing the opposite endogenous auxin dynamics during the two developmental pathways of the microspore, pollen development and microspore embryogenesis in *Brassica napus*. Green boxes illustrate changes during pollen development, and orange boxes changes during microspore embryogenesis, in IAA synthesis, accumulation, and expression of *TAA1* and *PIN1-like*.

The comparative study carried out indicate that endogenous auxin participates in the regulation of the two microspore developmental pathways, but would play different roles in each of them. During in vivo pollen development, auxin would have a relevant role in the early stages, associated with the high transcriptional activity of tapetum and microspores, decreasing with pollen maturation (Figure 9). However, endogenous auxin dynamics completely change with microspore reprograming to embryogenesis, showing an opposite profile that involves the increase in both auxin accumulation and the expression levels of the biosynthesis gene *TAA1* and the transport gene *PIN1* (Figure 9), which show an analogous pattern as described during zygotic embryogenesis. 

Taking all the results together, auxin would play a key role in the change of the developmental pathway of the microspore, where an opposite auxin dynamic determines the change of microspore cell fate, with auxin biosynthesis being required for microspore embryogenesis initiation and during subsequent embryo development.

## 4. Materials and Methods

### 4.1. Plant Material and Microspore Embryogenesis In Vitro Culture

*Brassica napus* L. (rapeseed) cv Topas line DH407 plants were used as donor plants. The seeds were germinated and grown under controlled conditions (relative humidity of 60%, photoperiod of 16 h light at 15 °C and 8 h dark at 10 °C) in a growth chamber (Sanyo MLR-351-H) in pots containing a mixture of organic substrate and vermiculite (2/1, *v*/*v*). Flower buds were collected to obtain anthers at different developmental stages and to isolate vacuolated microspores, the most responsive stage for embryogenesis induction. After isolation, microspore cultures were subjected to an in vitro stress treatment at 32 °C, as previously described [43], to induce embryogenesis.

### 4.2. Processing of Samples for Microscopy Analysis

Anthers and microspore culture samples were collected at different time points. Another samples were selected at the stages of tetrad, vacuolated microspore, and mature pollen. Microspore culture samples were chosen at the stages of isolated vacuolated microspore, proembryo, globular embryo, and coleoptilar embryo. The samples were fixed overnight at 4 °C using 4% paraformaldehyde in phosphate-buffered saline (PBS), washed with PBS, dehydrated in acetone series (30%, 50%, 70%, 90%, and 100%), and embedded in Technovit 8100 resin (Heraeus Kulzer GMBH, Hanau, Germany) at 4 °C. Semithin sections (2 µm thick) were collected on slides using an ultramicrotome (Ultracut E Reichert Jung, Eindhoven, The Netherlands). For cellular structure analyses, sections were stained with 1% toluidine blue in PBS, mounted with Eukitt, and observed under bright field microscopy. For immunofluorescence analyses, some sections were placed on aminopropyl-triethoxy-silane (APTES)-coated slides and stored at 4 °C until further use.

### 4.3. Immunofluorescence Assays and Confocal Microscopy Analysis

Immunofluorescence was carried out to localize indole-3 acetic acid (IAA) at different in vivo and in vitro developmental stages. The results were obtained from at least three immunofluorescence experiments with three biological replicates. Semithin sections were treated according to the following steps: blocking with 10% (*w/v*) fetal calf serum (FCS) in PBS for 10 min, incubating for 1 h with anti-IAA mouse monoclonal antibody (Sigma, cat. no. A0855) diluted 1:100 in 1% bovine serum albumin (BSA) in PBS, washing three times in PBS for 3 min each, incubating with Alexa Fluor 488–labeled anti-mouse IgG antibody (Molecular Probes-Invitrogen, Carlsbad, CA, USA) diluted 1:25 in 1% BSA (in darkness) for 45 min, and washing three times in PBS for 3 min each. Finally, sections were stained with 1 mg/mL DAPI (4.6-diamino-2-phenylindole) for 10 min, washed twice in distilled water for 5 min, mounted in Mowiol, and analyzed using confocal laser microscopy (Leica TCS-SP5-AOBS, Vienna, Austria). To ensure accurate comparison among immunofluorescence signal intensities of different developmental stages, confocal microscopy images were captured using the same laser excitation and sample emission settings in all immunofluorescence preparations. Maximum projection images were obtained from 10 optical sections (at 0.2 µm z-intervals) using the confocal microscope software (Leica software LCS version 2.5).

Negative controls were performed in two different ways: by omitting the primary antibody and by immunodepletion. This second negative control consisted of incubating the anti-IAA primary antibody with a 5 mg/mL IAA solution 1:2 (*v*/*v*) in PBS, overnight at 4 °C, to block the antibody with its antigen (IAA). Subsequently, this preblocked anti-IAA antibody was used as the primary antibody for immunofluorescence assays, following the same protocol as described above.

### 4.4. Quantification of Auxin Immunofluorescence Signal

The quantification of IAA immunofluorescence intensity was performed using the ImageJ software on confocal maximum projections, which were obtained as previously described. Regions of interest (ROIs) were outlined for structures at different developmental stages of anthers and microspore culture, and the fluorescence intensity values were obtained in arbitrary units. More than 20 structures from each developmental stage, obtained from over three different immunofluorescence experiments and at least three biological replicates, were measured. Significant differences among stages were tested by one-way ANOVA, followed by the Tukey’s multiple comparison test at *p* ≤ 0.05.

### 4.5. Quantification of Endogenous IAA Concentration

The quantification of endogenous IAA concentration was measured using HPLC coupled to tandem mass spectrometry as described in [81,82]. Briefly, samples of different developmental stages of microspore embryogenesis and pollen development were frozen in liquid nitrogen and ground to a fine powder using a prechilled mortar and a pestle. To proceed with total phytohormone extraction, 100 mg of powdered samples were extracted in ultrapure water using a tissue homogenizer (Ultra-Turrax, Merck, Darmstadt, Germany). Before extraction, samples were spiked with a standard solution composed of 100 ng of [^2^H_6_]-ABA, 100 ng of [^2^H_4_]-SA, and 100 ng of [^2^H_6_]-JA to assess recovery and matrix effects. After extraction and centrifugation, the pH of the supernatant was adjusted to 3.0 and partitioned twice against diethyl ether (Panreac, San Fernando de Henares, Spain). The organic layers were combined and evaporated in a centrifuge vacuum evaporator (Speedvac CS100, Savant, Thermo Fisher Scientific, Waltham, MA, USA). The dry residue was thereafter resuspended in a water/methanol (9:1) solution, filtered, and injected into an HPLC system (Alliance 2695, Waters Corp., Milford, CT, USA). Hormones were then separated in a reversed-phase Kromasil 100 C18 column (100 × 2.1 mm 5 μm particle size) using methanol and ultrapure water both supplemented with glacial acetic acid to a concentration of 0.05%. The mass spectrometer, a triple quadrupole (Quattro LC, Micromass Ltd., Manchester, UK), was operated in negative ionization electrospray mode, and plant hormones were detected according to their specific transitions using a multiresidue mass spectrometric method.

A minimum of three biological and three technical replicates were analyzed for each developmental stage. The final IAA concentration found in the analyses was determined by dividing the nanograms of hormone detected by the grams of fresh weight of the starting material. Differences among stages within each developmental process were tested by one-way analysis of variance (ANOVA), followed by Tukey’s multiple comparison test at *p* ≤ 0.05.

### 4.6. Expression Analysis by Reverse Transcriptase–Quantitative Polymerase Chain Reaction

Quantitative gene expression analyses were performed using qRT-PCR. Total RNA was extracted from anther and culture samples using the RNeasy^®^ Plant Micro and RNeasy^®^ Plant Mini kits (Qiagen Iberica, Las Rozas, Spain) and purified with NucleoSpin^®^ RNA Plant (Macherey-Nagel, Düsen, Germany), following the manufacturer’s instructions. cDNAs were obtained from 1.5 μg of RNA using the Superscript™ II Reverse Transcriptase (Invitrogen, Carlsbad, CA, USA) according to Solís et al. (2012). Quantitative real-time PCR was performed using the SsoAdvanced^TM^ Universal SYBR Green Supermix on the iQ™ 5 Real-Time PCR Detection System (Bio-Rad, Alcobendas, Spain). For the expression analyses of the *BnTAA1* gene, the following oligonucleotides were used: 5′-CGTCACCCAATAACCCAGAC-3′ and 3′-TCTTCCACCGTTTCTTCTAC-5′, from the sequence of the *BnTAA1* gene of *B. napus* (NCBI Reference Sequence: LOC103831591). For the expression analyses of the *BnPIN1-like* gene, the oligonucleotides used were as follows: 5′-CGACTCCCTCCAGAAAATCA-3′ and 3′-GACTTCGTGGTGCTAGACTT-5′, from the sequence of the *BnPIN1-like* gene of *B. napus* (NCBI Reference Sequence: LOC103831832). Serial dilutions of cDNA were prepared to stablish the efficiency curve of each primer pair. The thermocycling conditions were as follows: initial denaturation at 95 °C for 30 s, followed by 40 cycles of 5 s at 95 °C and 30 s at 58 °C. After each run, a dissociation curve was generated by heating the samples from 65 to 95 °C to verify amplification specificity. As internal control, the reference gene *BnActin* was selected, and the oligonucleotides used were 5′-TGAAGATCAAGGTGGTCGCA-3′ and 3′-GAAGATTCACAAAGACGGAAGA-5′ (BRAD Reference Sequence: Bra028615). A minimum of three biological and three technical replicates were included in the analyses. The data were analyzed using the Bio-Rad CFX Manager 3.1 (3.1.1517.0823) (Bio-Rad) software, employing the Livak calculation method [83]. Transcript levels were normalized to the tetrad stage in case of pollen development and to the proembryo stage in case of microspore embryogenesis. Statistical differences among stages were determined using one-way ANOVA, followed by Tukey’s multiple comparison test at *p* ≤ 0.05.

### 4.7. Treatments with Kynurenine and Evaluation of Its Effect in Microspore Embryogenesis

L-Kynurenine (Sigma), a small compound known for its inhibitory effects on endogenous auxin biosynthesis, was added to the culture media at the beginning of the in vitro microspore culture. For the assay, three different concentrations of the drug were tested: 4, 40, and 100 µM, added to the culture media by filtering a stock solution of 500 µM in DMSO (Sigma) using a sterile Minisart filter (Sartorius Biotech, Goettingen, Germany). In parallel, plates without the drug and with the corresponding volume of DMSO in each case were kept as control of the experiment (untreated cultures). The effects of the drug on embryogenesis initiation and progression were evaluated by quantifying the percentage of proembryos (the first morphological indication of embryogenesis initiation) and comparing the number of cotyledonary embryos formed after 30 days in culture between treated and control cultures. Mean percentages of proembryos were calculated from random micrographs obtained using a stereomicroscope in three independent experiments, with at least three different culture plates in each experiment. A minimum of 10,000 structures (proembryos and microspores) were counted for each condition. Statistical differences between untreated and treated cultures at different concentrations of the drug were determined using ANOVA, followed by Tukey’s multiple comparison test at *p* ≤ 0.05. Images of the plates after 30 days of culture were captured using a Nikon D810 camera to evaluate the production of cotyledonary embryos formed after 30 days.

## Data Availability

The data presented in this study are available on request from the corresponding author.
